# Incremental cost and health gains of the 2016 WHO antenatal care recommendations for Rwanda: results from expert elicitation

**DOI:** 10.1186/s12961-019-0439-9

**Published:** 2019-04-05

**Authors:** Regis Hitimana, Lars Lindholm, Ingrid Mogren, Gunilla Krantz, Manasse Nzayirambaho, Jean-Paul Semasaka Sengoma, Anni-Maria Pulkki-Brännström

**Affiliations:** 1 School of Public Health, College of Medicine and Health Sciences, University of Rwanda, Kigali, Rwanda; 2 Department of Epidemiology and Global Health, Umeå University, SE 901 87 Umeå, Sweden; 3 Department of Clinical Sciences, Obstetrics and Gynecology, Umeå University, 901 87 Umeå, Sweden; 4 Department of Public Health and Community Medicine, The Sahlgrenska Academy, University of Gothenburg, Gothenburg, Sweden

## Abstract

**Objectives:**

High-quality evidence of effectiveness and cost-effectiveness is rarely available and relevant for health policy decisions in low-resource settings. In such situations, innovative approaches are needed to generate locally relevant evidence. This study aims to inform decision-making on antenatal care (ANC) recommendations in Rwanda by estimating the incremental cost-effectiveness of the recent (2016) WHO antenatal care recommendations compared to current practice in Rwanda.

**Methods:**

Two health outcome scenarios (optimistic, pessimistic) in terms of expected maternal and perinatal mortality reduction were constructed using expert elicitation with gynaecologists/obstetricians currently practicing in Rwanda. Three costing scenarios were constructed from the societal perspective over a 1-year period. The two main inputs to the cost analyses were a Monte Carlo simulation of the distribution of ANC attendance for a hypothetical cohort of 373,679 women and unit cost estimation of the new recommendations using data from a recent primary costing study of current ANC practice in Rwanda. Results were reported in 2015 USD and compared with the 2015 Rwandan per-capita gross domestic product (US$ 697).

**Results:**

Incremental health gains were estimated as 162,509 life-years saved (LYS) in the optimistic scenario and 65,366 LYS in the pessimistic scenario. Incremental cost ranged between $5.8 and $11 million (an increase of 42% and 79%, respectively, compared to current practice) across the costing scenarios. In the optimistic outcome scenario, incremental cost per LYS ranged between $36 (for low ANC attendance) and $67 (high ANC attendance), while in the pessimistic outcome scenario, it ranged between $90 (low ANC attendance) and $168 (high ANC attendance) per LYS. Incremental cost effectiveness was below the GDP-based thresholds in all six scenarios.

**Discussion:**

Implementing the new WHO ANC recommendations in Rwanda would likely be very cost-effective; however, the additional resource requirements are substantial. This study demonstrates how expert elicitation combined with other data can provide an affordable source of locally relevant evidence for health policy decisions in low-resource settings.

**Electronic supplementary material:**

The online version of this article (10.1186/s12961-019-0439-9) contains supplementary material, which is available to authorized users.

## Introduction

In evidence-informed health policy, meta-analysis of randomised controlled trials is considered to be at the top of the hierarchy of good evidence, whereas evidence from specialists and clinical practice is considered to be at the bottom [1]. Internationally published health research is of limited local relevance for low-income countries, given that most of the evidence is generated from high-income countries [2]. Many systematic reviews do not reflect the priorities and problems of low-resource settings [2, 3], and even when the reviews are relevant, the interventions and treatments suggested may be unavailable, unaffordable or inappropriate for the setting [2].

One example in practice are the new recommendations on antenatal care (ANC) for improved pregnancy outcomes issued by WHO in November 2016 [4], which replace the 2002 Focused ANC model [5]. The major new features of the 2016 WHO recommendations are eight ANC contacts during pregnancy and one obstetric ultrasound examination before gestational week 24 for all pregnant women. The ultrasound recommendation is based on a Cochrane systematic review from 2015 that includes 11 randomised controlled trials on populations from six countries (Australia, Norway, South Africa, Sweden, the United Kingdom and the United States of America) [4, 6]; no low- or lower- to middle-income countries were included in this review.

Rwanda’s current ANC policy was developed based on the 2002 WHO recommendations, which suggest four focused ANC visits for normal pregnancy [7]. In 2014/2015 only 45% of pregnant women attended the full four ANC visits, and the first visit was only in the fifth month of gestation on average [8]. Maternal mortality in Rwanda has been significantly reduced over the last 15 years from 1071 per 100,000 live-births in 2000 to 210 per 100,000 live births in 2014/2015 [8, 9]. This is still three times higher than the global target for maternal mortality in the Sustainable Development Goals, namely to reduce the global maternal mortality ratio to less than 70 per 100,000 live births [10]. In its strategy 2018–2024, Rwanda aims to reduce the maternal mortality ratio to 126 per 100,000 live births [11].

Decision-makers constrained with insufficient budgets need evidence from health economic evaluations to make informed decisions about the efficient use of resources for meeting population health needs [12]. Economic evaluations help to identify, measure and compare the cost and health impacts of competing health interventions as well as their scalability and sustainability [12]. However, the use of economic evaluation is still limited in low- and middle-income countries [13]. One reason is the unavailability and limited local relevance of published research, which calls for alternative sources of evidence to be used. One method increasingly used in such situations is expert elicitation, which can be used to collect quantitative and qualitative information such as proportions, percentages and probabilities of events [14].

The aim of this study was to inform decision-making on ANC recommendations in Rwanda by estimating the incremental cost and health gains of implementing the 2016 WHO recommendations compared to current practice.

## Methods

### Data sources

The intervention of interest was ANC for normal (uncomplicated) pregnancies in the public healthcare system in Rwanda. The cost and health outcomes of current ANC practice were compared with a hypothetical situation in which the 2016 WHO ANC recommendations are adopted and implemented over a 1-year time period. The study was a (prospective) simulation-based cost effectiveness study for a hypothetical cohort of 373,679 women, equal to the number of women who attended ANC at least once in Rwanda in the year 2014 [15].

The unit cost of current ANC practice, and current patterns of ANC attendance, were obtained from two sub-studies of Maternal Health Research in Rwanda (MatHeR), a research programme comprising cross-sectional health facility and household surveys conducted in Rwanda in 2014 [16, 17]. Incremental resource use was identified and quantified by comparing the key activities during each of the eight visits detailed in the 2016 WHO ANC recommendations [4], with resource use under the current four-visit model [16]. Primary data was collected using expert elicitation to estimate health gains in terms of mortality decreases as detailed below. Health outcomes were expressed as life-years saved (LYS) using current mortality from the 2015 Annual Health Statistical Booklet for the Rwandan Ministry of Health [18] and life expectancy from the report of the Rwanda Fourth General Population and Housing Census 2012 [19]. Incremental cost-effectiveness ratios were estimated from the societal perspective comprising health sector and household costs.

A list of all data sources, the kind of information obtained, and the use of that information are presented in Additional file 1.

### Cost measurement

Incremental cost was estimated in three steps. First, assumptions were made about the unit cost of each of the eight ANC visits under the 2016 WHO recommendations. Second, the distribution of ANC attendance was simulated in the hypothetical situation that Rwanda implements the new recommendations, and the cost of ANC was obtained for each pregnant woman in the hypothetical cohort. Third, incremental total cost and incremental cost per woman were computed as the difference between average cost from the simulation and the average cost of current ANC practice. Each of these steps is described in detail below.

#### Assumptions about unit cost

Assumptions about the unit cost of the eight visits were based on our previous study of the unit cost of the four ANC visits that women with normal (uncomplicated) pregnancies are expected to complete under the current ANC policy in Rwanda [16]. That study used a micro-costing approach to collect and analyse resource use by healthcare providers and households from eight public and faith-based health facilities in the Northern province and in Kigali city in Rwanda.

The main changes implied by the 2016 WHO ANC recommendations compared to current practice in Rwanda that have considerable resource use implications are (1) an increase in the number of visits from four to eight contacts during pregnancy; (2) one early obstetric ultrasound examination before week 24 of gestation, in place of selective ultrasound only; and (3) repetition of some laboratory tests in late pregnancy. Based on the above changes, the following assumptions related to incremental changes in the unit cost were made:

Firstly, the cost of an ultrasound examination, estimated at $3 [16], was added to the cost of the first ANC visit. Neither the WHO recommendation nor the systematic review of trials that underpinned it [20] specified whether the ultrasound should be conducted during the first trimester (Visit 1) or early in the second trimester, as long as it is before week 24 of gestation. Because women in Rwanda typically register late for ANC, and to simplify the calculations, we added the cost of ultrasound to Visit 1. Otherwise, the cost of the first visit was assumed to be equal to current cost ($21), thus including a number of laboratory tests and a mosquito net. Secondly, Visits 2, 4, 5 and 7 in the new model were taken to be ordinary routine ANC visits, and therefore equal in cost to Visits 2 and 3 in the current model ($6). Third, Visits 3 and 6 in the new model were assumed to be routine visits but with the addition of two repeat laboratory tests, namely tests for bacteriuria and for anaemia, for which costs were estimated at $3 and $0.75, respectively [4, 21]. The last visits in the two models were assumed to be equal in cost ($11). The summary of the unit cost in the currently implemented four-visit model and unit cost estimates in the new eight-visit model are presented in Table 1.

**Table 1:** Comparison between unit cost of ANC services in the current four-visit model and assumptions about unit cost in the eight-visit model (2015 USD)

Four-visit model		Eight-visit model	
ANC visit	Unit cost ($)	ANC visit	Unit cost ($)
Visit 1	21	Visit 1	24
Visit 2	6	Visit 2	6
Visit 3	6	Visit 3	10
Visit 4	11	Visit 4	6
		Visit 5	6
		Visit 6	10
		Visit 7	6
		Visit 8	11

#### Simulation of ANC attendance

Three costing scenarios, corresponding to different assumptions about ANC attendance, were simulated using R software (version 3.3.2, The R Foundation for Statistical Computing). The assumptions were based on current attendance patterns in Rwanda, the schedule suggested in the 2016 WHO ANC recommendations and previous literature on ANC attendance in other countries.

ANC attendance under the current ANC policy in Rwanda has been previously reported using a household survey conducted in 2014 [17]. The analysis of the dataset, to which we had access, showed that the mean number of ANC visits is 3.3 per woman, with a median of 3 and a standard deviation of 0.8. A histogram of the distribution, which approximates a normal distribution, is provided in Additional file 2.

Given the current distribution, it is unlikely that if Rwanda implements the 2016 WHO ANC recommendations, the average number of visits becomes eight per woman as specified in the recommendations, at least in the short to medium term. This assumption is further corroborated by findings in the Cochrane review [22] and the WHO ANC trial [23] that, while women in high-income countries generally complete at least the minimum number of ANC visits recommended by the policy in place, in low- and middle-income countries, women generally do not reach the recommended number of ANC visits. One reason is late registration in ANC [22], as is the case in Rwanda and the region [24–29]. A summary of ANC attendance in the studies included in the Cochrane systematic review is presented in Additional file 3.

The scenarios were generated using Monte Carlo simulation, a numerical technique that assists in generating random numbers and forces them to follow a pre-determined probability distribution [30]. The hypothetical cohort was 373,679 women, equal to the number of women who attended ANC at least once in Rwanda in 2014 [15]. Since the true distribution is unknown, the mean and standard deviation were used to simulate a normal distribution [31]. The simulations resulted in the following three scenarios of ANC attendance in the new eight-visit model. In the ‘conservative’ Scenario 1, the mean number of ANC visits per woman is five; a pregnant woman who attends the minimum number of visits, attends only once; and the maximum number of visits is eight. In Scenario 2, the mean number of visits is six, the minimum is two, and the maximum number is 10. In the ‘ambitious’ Scenario 3, the mean number of visits is seven, the minimum number is three, and the maximum number is 11. Histograms of the distributions of the three attendance scenarios are presented in Additional file 2.

For the purpose of presenting alternative policy options, a scenario of full compliance to the current ANC guidelines was also constructed, in which all women attend the full four ANC visits.

#### Incremental cost estimation

Incremental cost was estimated in four main steps. First, the cost of ANC was calculated for each member of the 373,679 cohort by multiplying the simulated number of ANC visits for each individual by their respective unit costs. For example, the cost of ANC for a woman who attends five visits was calculated as the sum of Visits 1–5 in Table 1. Second, the total cost of ANC in the simulation was calculated by summing up the cost of ANC for all individuals. This procedure was repeated 100 times and differences between the minimum and maximum were compared to check for reliability of the simulation. Third, the average total cost was used to compute average cost of ANC per woman. Finally, incremental cost was computed by comparing each of the four costing scenarios with the cost of current ANC practice.

### Health outcome measurement

Two health outcomes scenarios (optimistic, pessimistic) were constructed to estimate the incremental health gains from the 2016 WHO recommendations compared to current practice. First, changes expected in maternal and perinatal mortality were estimated using expert elicitation. Second, the resulting mortality reductions were transformed into deaths averted and life-years saved using secondary data.

#### Expert elicitation

Expert elicitation using aspects of the Delphi process was used to estimate how implementing the 2016 WHO ANC recommendations in Rwanda would potentially affect perinatal and maternal mortality. The respondents were selected using the inclusion criteria of having specialist training in obstetrics and to have practiced in Rwanda for at least 5 years after training. A total of 70 gynaecologists/obstetricians registered in Rwanda were identified, of which 19 fulfilled the inclusion criteria. All 19 were invited for interview by e-mail (with the questionnaire as an attachment; Additional file 4). One week later, an appointment was proposed via telephone. Three specialists declined the invitation either due to time constraints or travel. Two other specialists agreed to participate but were not available for interview within the specified 3-week timeframe. Six specialists did not respond to the invitation. Finally, eight specialists were interviewed in April 2017.

The interviewer-administered questionnaire was composed of three main parts. The first part described trends in maternal and newborn health with key indicators in Rwanda and changes to ANC practice proposed by the new 2016 WHO ANC recommendations. The second part included questions about the potential implications of the new recommendations on cause-specific and overall perinatal and maternal mortality. The third part invited the respondents to suggest improvements to ANC practice other than implementing the 2016 WHO ANC recommendations in full, and to estimate the possible implications on perinatal and maternal mortality.

According to WHO, a maternal death is “*the death of a woman while pregnant or within 42 days of termination of pregnancy, irrespective of the duration and site of the pregnancy, from any cause related to or aggravated by the pregnancy or its management but not from accidental or incidental causes*” [32]. While perinatal mortality has different definitions [33], according to WHO, the perinatal period commences at 22 completed weeks of gestation and ends 7 completed days after birth. Perinatal mortality refers, therefore, to deaths of the fetus from 22 completed gestational weeks (154 days) and deaths in the first week of life (7 days) [34].

The first draft of the questionnaire was developed by the first author and revised based on comments from the multi-disciplinary co-authors’ team, composed of health economists, obstetricians and public health researchers. All interviews were conducted by the first author. The questionnaire is presented in Additional file 4.

After an initial analysis of the responses, it was decided to give respondents an opportunity to re-evaluate their responses after examination of the group response, following the principles of the Delphi process [35]. A second round of opinion collection was therefore conducted in July–August 2017. The eight respondents were re-contacted by email and phone and presented with the anonymised group responses. Five specialists revised their estimates, two kept their initial estimates, and one did not reply. In the latter case, non-response was taken as confirmation of the respondent’s initial estimates. Each respondent’s estimates of the maternal and perinatal mortality changes from the two rounds are presented in Additional file 5.

Responses from the second round were retained as inputs in the analysis. Two outcome scenarios were constructed by ordering responses to each of the two indicators from low to high and dividing into two equal sub-groups labelled either the ‘optimistic’ health outcome scenario, composed of relatively higher reductions in mortality, or the ‘pessimistic’ health outcome scenario, composed of relatively lower reductions in maternal and perinatal mortality. The mean response in each sub-group was retained as the parameter estimate for each scenario.

#### Estimation of life-years saved (LYS)

The estimated reductions in mortality were converted into deaths averted over a hypothetical 1-year implementation period in order to summarise the two mortality figures in one unit of measurement. The number of deaths averted was calculated using death counts from the 2015 Annual Health Statistical Booklet for the Rwandan Ministry of Health [18]. In 2015, maternal and perinatal mortality deaths were estimated at 371 and 10,076, respectively [18]. Then, maternal LYS were calculated using proportions of maternal mortality by age groups from the Demographic and Health Survey 2014/2015 report [8], life expectancy at birth for women (66 years) for life-years of mothers, and life expectancy at birth for both sexes (64 years) for perinatal life-years, from the 2012 Rwanda Census [19]. LYS were discounted a 3% rate (Additional file 6).

### Cost effectiveness

Incremental cost effectiveness of the 2016 WHO ANC recommendations compared to current ANC practice in Rwanda was expressed as cost per LYS for each of the three costing scenarios (reflecting different attendance assumptions) and the two health outcome scenarios.

No official or country-specific estimate of the cost effectiveness threshold is currently available for Rwanda. Thresholds based on Gross Domestic Product (GDP), previously recommended by WHO [36] in cases where there is no official threshold, were therefore used. Any intervention that averts a disability-adjusted life-year for the cost of a given country’s annual GDP per capita or less is considered to be ‘very cost effective’, and an intervention is labelled as not cost effective if the cost is more than three times per capita GDP [37]. The Rwandan GDP per capita was $697 in (2015 USD) [38].

## Results

### Cost of the 2016 WHO ANC recommendations in Rwanda

The total and incremental cost of the 2016 WHO ANC recommendations from the 100 simulations for each of the three attendance scenarios is presented in Table 2. The results suggest that, on average, the cost of ANC per woman ranges between $53 and $69 (standard deviation $7). The results further suggest that, on average, the total national cost of ANC ranges between $19.8 million and $24.9 million. The difference between minimum and maximum values for each scenario across the simulations are 0.001% and below, which indicates that the simulation model is reliable.

**Table 2:** Incremental cost of the 2016 WHO ANC recommendation for Rwanda ($2015)

Costing scenario	Cost per woman		Total cost			Incremental cost	
	Mean,	SD	Mean	Min	Max	Per woman	Total
Current practice	37		13,939,970				
Scenario 1 ‘conservative’	53	6.6	19,829,260	19,818,549	19,837,028	9	5,889,290
Scenario 2	61	6.9	22,799,062	22,789,107	22,811,818	17	8,859,092
Scenario 3 ‘ambitious’	69	6.8	24,895,023	24,873,568	24,917,497	25	10,955,053
Full utilisation current policy	44	4	16,441,832	–	–	–	2,501,862

Notes to Table 2: Current practice from [16]. Scenarios 1–3 are results from Monte Carlo simulation with differing mean, minimum and maximum number of visits as specified in Methods

The average cost of current ANC practice has previously been reported to be $44 per woman, and the total national cost to be $13.9 million [16]. The incremental total cost of the 2016 WHO ANC recommendations ranges therefore between $5.9 million and $11 million across the attendance scenarios (Table 2). The costs increase by 42%, 64% and 79% compared to current practice in Scenarios 1–3, respectively. Full utilisation under the current policy would increase costs by 18%.

### Life-years saved (LYS)

Implementation of the 2016 WHO ANC guidelines was estimated to reduce perinatal mortality by 22.5% and 55% in the pessimistic and optimistic health outcome scenarios, respectively, equivalent to 2267 and 5542 perinatal deaths avoided per year (Table 3). The estimated reduction in maternal mortality was 7% and 52% in the pessimistic and optimistic scenarios, respectively, representing 26 and 195 maternal deaths avoided. In total, the incremental health gains were 65,366 LYS in the pessimistic scenario and 162,509 LYS in the optimistic scenario, of which 99% and 98%, respectively, were due to perinatal deaths avoided.

**Table 3:** Incremental health benefits of the 2016 WHO ANC recommendations compared to current practice in Rwanda

Mortality measure	Mortality (DHS 2014/2015)	Annual deaths (DHS 2014/2015)	Mortality reduction			Life-years saved
			Outcome scenario	% change	Avoided deaths	
Perinatal mortality	29 per 1000 pregnancies	10,076	Pessimistic	−22.50%	2267	64,827
			Optimistic	−55%	5542	158,466
Maternal mortality	210 per 100,000 live-births	371	Pessimistic	−7%	26	539
			Optimistic	−52.50%	195	4044

### Incremental cost-effectiveness

Incremental costs per LYS from implementation of the 2016 WHO ANC recommendations in different cost and outcome scenarios are presented in Table 4. Results indicate that incremental cost per LYS ranges between $36 and $67 in the optimistic outcome scenario and between $90 and $168 in the pessimistic scenario. Thus, implementing the new recommendations in Rwanda would be very cost-effective in all the scenarios presented in this study.

**Table 4:** Incremental cost per life-year saved (in USD)

Costing scenario	Incremental cost	Cost per life-year saved	
		Optimistic outcome scenario	Pessimistic outcome scenario
Scenario 1 ‘conservative’	$5,889,290	$36	$90
Scenario 2	$8,859,092	$55	$136
Scenario 3 ‘ambitious’	$10,955,053	$67	$168

Notes to Table 4: The number of life-years saved is 162,509 in the optimistic and 65,366 in the pessimistic outcome scenario

## Discussion

This study analyses the incremental cost-effectiveness of the 2016 WHO ANC recommendations for Rwanda using evidence from expert elicitation, simulation of attendance patterns, and estimation of their unit cost implications. The results indicate that implementing the new 2016 WHO ANC recommendations in Rwanda would likely be very cost-effective. However, the additional resource requirements are significant for both the health sector and for households. The $5.8 million, $8.8 million and $11 million in incremental costs for the three costing scenarios represent 3%, 5% and 6% of the 2017/2018 health sector budget ($183.1 million) [39], respectively. The 2016 WHO recommendations include doubling the number of ANC visits per woman with an uncomplicated pregnancy, adding some new activities, in particular one routine obstetric ultrasound, and repeating some laboratory tests. Our results suggest that the new recommendations do not imply doubling the cost of the currently implemented four-visit model. The main explanation is that the first visit, which represents nearly half of the cost of the current four-visit schedule, remains by far the most costly visit also in the eight-visit schedule. For households, the cost of ANC is likely to increase nearly in proportion to the number of visits. Although this study did not separately report the relative importance of households and health sector, the household contribution was representing 9% of the total societal cost of current ANC practice in Rwanda [16].

The findings of this study suggest large health gains, ranging between 65,366 and 162,509 LYS per year of implementation, mostly due to a reduction in perinatal mortality. These results from expert elicitation are consistent with previous literature that suggests the critical changes suggested by the new recommendations (such as doubling the number of ANC visits and a routine ultrasound examination before 24 weeks) are associated with saving babies’ rather than mothers’ lives [40–42]. The maternal health package is composed of a set of interdependent interventions, for which the relative effectiveness of each element of the package is hard to measure [43]. Maternal deaths occur mainly during labour, delivery and in the immediate postpartum, and postpartum haemorrhage, sepsis and other direct causes together represent close to the half of the causes of maternal mortality [44]. Therefore, while it has been estimated that ANC can save 26% of maternal deaths in low-resource settings, saving women’s lives depends to a larger extent on the quality of obstetric care rather than ANC [45, 46]. Other explanations for the relative emphasis on perinatal rather than maternal LYS in the results are differences in the current level of mortality and the larger relative weight associated with a perinatal death in the LYS computation. Together, these imply that a given percentage reduction in perinatal mortality is equivalent to a relatively larger number of LYS than the same reduction in maternal mortality.

Current data on population health and the cost of care is increasingly becoming available and locally relevant even in low-resource settings. However, scientifically rigorous methods are still rarely applied to use that evidence to inform health policy decision-making. In this study, we combined primary costing and attendance data with results from simulation and expert elicitation to estimate the implications on cost and health outcomes of a change in current ANC policy suggested by new WHO recommendations. Expert elicitation is increasingly used in healthcare research, specifically in model-based health economic evaluations [47, 48] and in developing evidence-based clinical guidelines [49]. It is used to complement information from other type of evidence especially when data are unavailable, expensive or difficult to collect [14] or when the available data are not relevant. Current limitations to using expert elicitation include limited guidance on how to successfully conduct expert elicitation [50], and how to deal with cognitive heuristics and biases [51]. In this study, we provided respondents with references to the available literature on common causes of perinatal and maternal mortality in Rwanda and in low-income countries, in order to limit bias by overlooking pertinent evidence. However, the small number of experts who participated in the expert elicitation (*n* = 8) is one of the limitations of this study. It was mainly due to limited number of active practitioners who fulfil the inclusion criteria. We believe that expert elicitation has contributed by adding scientific rigor to the ongoing policy debate and demonstrated its potential as an alternative solution for evidence-informed decision-making.

Further research is needed on the mortality effects of the new recommendations but also on the relationship between ANC attendance and health benefits, especially in low-income countries where mortality is still high. At present, the available evidence is insufficient to create combined scenarios of attendance and health outcomes, which we dealt with in this study by allowing combinations that may be considered unlikely, such as the combination of the high cost (attendance) with the pessimistic outcome scenario. Another limitation is that this study limited the consideration of health gains to maternal and perinatal mortality. In a previous study, we found evidence that health-related quality of life 1 year postpartum was positively associated with adequate ANC utilisation [52]. Other studies have similarly shown that the benefits of ANC extend beyond mortality to a reduction in maternal morbidity, women’s life satisfaction [4], children’s health status [6], and control of other diseases such as malaria and HIV. Increased use of ANC can affect maternal morbidity in two ways – first, regular exposure to some ANC interventions can reduce the risk of certain health conditions during pregnancy; for example, there is evidence suggesting that daily iron supplementation reduces the risk of maternal puerperal sepsis [53]. Secondly, screening for existing maternal health conditions such as anaemia, HIV, syphilis, malaria, etc., reduces their burden on mothers who regularly attend ANC services, through increased chances for early detection and management, especially in high prevalence settings [4]. Health promotion activities included in the WHO ANC recommendations like hygiene, nutrition, and physical activity, can also be translated into better nutrition and hygiene for other children.

The method used in this study to compute total cost of ANC per woman by adding up the unit cost of ANC visits according to the schedule might not reflect reality, in particular when the number of visits falls well below the recommended number. For example, a pregnant woman who comes for her second ANC visit close to delivery, in effect receives Visit 8, instead of Visit 2, as has been assumed here. Furthermore, the 2016 WHO recommendations have other cost implications for Rwanda that have not been explored in this analysis. For example, the large increase in the number of ANC visits is likely to replace other health facility services. On the other hand, there may be some economies of scale, i.e. the same infrastructures are used by more people and consequently at lower cost per woman. Economies of scope could also be expected if, for example, ultrasound equipment could be used for other purposes such as gynaecological examinations. There will likely be increased need for supervision and training of staff; however, this analysis did not consider any one-off costs, such as ultrasound training, or the procurement of new ultrasound machines. Currently in Rwanda, obstetric ultrasound is only available in hospitals and performed by physicians [33, 54, 55]. There are disparities between rural and urban health facilities in terms of the availability and quality of ultrasound equipment [33, 55]. Lastly, the projection of cost and health gains from an actual future policy change would require minor adjustments to the results presented here because prices, attendance, mortality and life expectancy data refer to figures collected in 2014–2015.

## Conclusion

Implementation of the new 2016 WHO ANC recommendations has the potential to cost-effectively reduce perinatal and maternal mortality in Rwanda. Based on these findings, it can be concluded that the Government should consider implementation of these recommendations. However, given the significant investment required for implementation, and a narrow fiscal space, an alternative is that the health sector adopts a phased approach to implementation, starting with the most feasible activities within the 2016 WHO ANC recommendation. This could imply, for example, increasing the recommended number of visits to some level that can be managed with current staffing levels. Nevertheless, there is also a need to investigate the reasons behind the low attendance to ANC within the current ANC policy and to address them, and hence, preparing the ground for an expanded ANC package. Our study also demonstrates that expert elicitation can be successfully combined with other sources of data to support health policy decision-making, especially when high-quality evidence of effectiveness and cost effectiveness is not available or locally relevant.

## Additional files

Additional file 1: Summary of data sources used. (DOCX 21 kb)

Additional file 2: Distribution of women according to the number of antenatal care visits attended, in the current four-visit model. (DOCX 42 kb)

Additional file 3: Summary of antenatal care utilisation in different countries from the Cochrane systematic review of antenatal care and WHO trial. (DOCX 14 kb)

Additional file 4: Questionnaire for collection of opinions on potential health outcomes that can be expected from the expansion of antenatal care programme in Rwanda. (DOCX 38 kb)

Additional file 5: Calculation of incremental cost. (XLSX 34 kb)

Additional file 6: Calculation of discounted Life Years Saved. (XLSX 33 kb)

## Abbreviations

ANC: antenatal care
GDP: gross domestic product
LYS: life-years saved
SD: standard deviation
