# Supplementary material for: Incremental cost and health gains of the 2016 WHO antenatal care recommendations for Rwanda: results from expert elicitation
Source: Health Res Policy Syst. 2019 Apr 5;17:36. doi: 10.1186/s12961-019-0439-9 (PMC6451275; doi:10.1186/s12961-019-0439-9)
Supplement: Supplementary file 1 — Summary of data sources used. (DOCX 21 kb) [file 12961_2019_439_MOESM1_ESM.docx]

**Additional file 1: Summary of data sources used**

| **No** | **Data source** | **Information** | **Used for** |
| --- | --- | --- | --- |
| 1 | Hitimana et al. 2017(1) | Cost estimation from 5 selected health facilities in Rwanda (2015) | - Making assumption of unit costs  - As baseline for estimation of incremental cost |
| 2 | Rurangirwa et al. (2017) (2) | Distribution of ANC attendance in Rwanda in 2014 | - Distribution parameters for ANC attendance used in simulation of the future attendance of ANC |
| 3 | WHO ANC recommendations 2016 (3) | Schedule of ANC visits and activities per visit | -Estimation of incremental cost  - Background information for experts |
| 6. | Results of expert elicitation (Additional file 5) | Maternal and perinatal mortality changes | Used in calculation of YLS |
| 4 | Rwanda Demographic and Health Survey Report 2014/2015(4) | Perinatal mortality and age specific maternal mortality | -Estimation of Years of life saved |
| 5. | Rwanda fourth General population and housing census, 2012 (5) | Life expectancy at birth | Used in calculation of life years lost |

**REFERENCE**

1. Hitimana R, Lindholm L, Krantz G, Nzayirambaho M, Pulkki-Brännström A-M. Cost of antenatal care for the health sector and for households in Rwanda. BMC Health Services Research [Internet]. 2018 Apr;18(1):262. Available from: https://doi.org/10.1186/s12913-018-3013-1

2. Rurangirwa AA, Mogren I, Nyirazinyoye L, Ntaganira J, Krantz G. Determinants of poor utilization of antenatal care services among recently delivered women in Rwanda ; a population based study. BMC Pregnancy and Childbirth; 2017;1–10.

3. World Health Organization. WHO Recommendation on Antenatal care for positive pregnancy experience. WHO Recommendation on Antenatal care for positive pregnancy experience [Internet]. 2016;152. Available from: http://apps.who.int/iris/bitstream/10665/250796/1/9789241549912-eng.pdf

4. National Institute of Statistics of Rwanda (NISR) [Rwanda], Ministry of Health (MOH) [Rwanda] and I, International. Rwanda Demographic and Health Survey 2014-15. 2015.

5. National Institute of Statistics of Rwanda (NISR). Fourth Population and Housing Census, Rwanda, 2012. Rwanda. 2012;226(4676):782.
