# Supplementary material for: Incremental cost and health gains of the 2016 WHO antenatal care recommendations for Rwanda: results from expert elicitation
Source: Health Res Policy Syst. 2019 Apr 5;17:36. doi: 10.1186/s12961-019-0439-9 (PMC6451275; doi:10.1186/s12961-019-0439-9)
Supplement: Supplementary file 2 — Distribution of women according to the number of antenatal care visits attended, in the current four-visit model. (DOCX 42 kb) [file 12961_2019_439_MOESM2_ESM.docx]

**Additional file 2: Distribution of women according to the number of antenatal care visits attended, in the current 4-visit model.**

**Distributions of suggested three scenarios of antenatal care utilization**

**
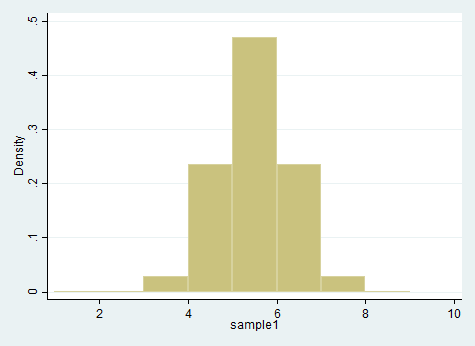
**

mean=5, sd=0.8

**
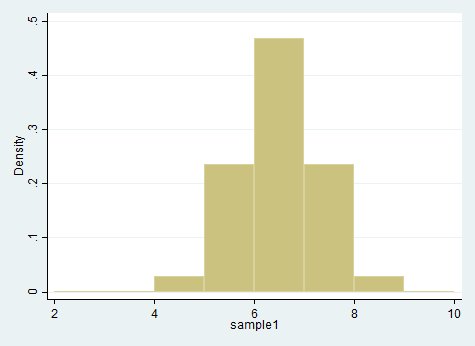
**

mean=6, sd= 0.8

**
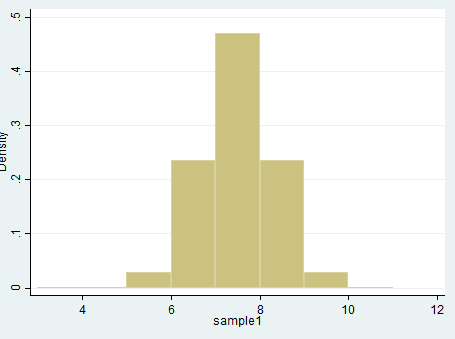
**

mean=7, sd = 0.8
