# Supplementary material for: Incremental cost and health gains of the 2016 WHO antenatal care recommendations for Rwanda: results from expert elicitation
Source: Health Res Policy Syst. 2019 Apr 5;17:36. doi: 10.1186/s12961-019-0439-9 (PMC6451275; doi:10.1186/s12961-019-0439-9)
Supplement: Supplementary file 3 — Summary of antenatal care utilisation in different countries from the Cochrane systematic review of antenatal care and WHO trial. (DOCX 14 kb) [file 12961_2019_439_MOESM3_ESM.docx]

**Additional file 3: Summary of antenatal care utilization in different countries from the Cochrane systematic review of antenatal care and WHO trial**

| **Study** | **Group 1: Reduced** | | **Group 2: Standard** | |
| --- | --- | --- | --- | --- |
|  | **Planned** | **Achieved** | **Planned** | **Achieved** |
| England 1996 | 7 | 8.6 | 13 | 10.8 |
| USA 1995 | 8 | 8.2 (SD 1.9) | 13 | 11.3 (SD: 2.1) |
| USA 1996 | 9 | 12 (SD 4.2) | 14 | 14.7 (DS 4.2) |
| USA 1997 | 8 | 7.6 (SD 1.6) | 14 | 10.8 (SD 2.3) |
| Zimbabwe 1996 | 6 | 4 | 14 | 6 |
| Sweden 1998 |  | 12 (SD 2.75) |  | 10.45 (2.6) |
| London 1996 | 7 | 8.6 (2-77) | 13 | 10.8 (3-01) |
| Sweden 2005 |  | Prim 11.1 (SD 4.5) 2.9 (SD 3.0) to physician |  |  |
|  |  | Multi 9.2 (SD 3.6) and 2.7  (SD 2.9) physician |  |  |
| **WHO trial (mean value and interquintiles)** | | | | |
| Argentina |  | 5 [3-6] |  | 7 [4-9] |
| Cuba |  | 8 [5-10] |  | 13 [12-14] |
| Thailand |  | 3.5 [2-4] |  | 6 [4-8] |
| Saudi Arabia |  | 3.5 |  | 4.5 |
