# Supplementary material for: Incremental cost and health gains of the 2016 WHO antenatal care recommendations for Rwanda: results from expert elicitation
Source: Health Res Policy Syst. 2019 Apr 5;17:36. doi: 10.1186/s12961-019-0439-9 (PMC6451275; doi:10.1186/s12961-019-0439-9)
Supplement: Supplementary file 4 — Questionnaire for collection of opinions on potential health outcomes that can be expected from the expansion of antenatal care programme in Rwanda. (DOCX 38 kb) [file 12961_2019_439_MOESM4_ESM.docx]

**Additional file 4: Questionnaire for collection of opinions on potential health outcomes that can be expected from the expansion of antenatal care program in Rwanda.**

**Identification of interviewee**

**Names:**

**Sex:**

**Employer:**

**Experience (in years) as Obstetrician:**

**Background**

Over the last two decades, Rwanda has made tremendous progress in reducing maternal and infant mortality from 1071 to 210 maternal deaths per 100,000 live births and from 107 to 32 infant deaths per 1,000 live births respectively(1)(2). Those improvements have been attributed to improvements in demand and supply of key interventions including antenatal care, skilled delivery as well as health system reforms including community health program and community based health insurance (3).

As you know, Rwanda has adopted the World Health Organization (WHO) antenatal care model composed of 4 visits: one visit in the first trimester, one in the second trimester, and two in the third). Our research conducted in 2014 suggested that almost all women (99,5%) attended at least one antenatal visit but only 44,5% visited antenatal care (ANC) the recommended four times or more. These figures are similar to those from the Demographic and Health Survey 2014/15.

In November 2016, WHO has published new recommendations for antenatal care. The main changes compared to current recommendations in Rwanda are:

- Increase in the number of visits from 4 visits to 8 visits. In the first trimester 1 visit, second trimester 2 visits, third trimester 5 visits
- Introduction of ultrasound before week 24 of pregnancy
- Nutritional counseling 8 times
- Test for anemia (3 times) and for asymptomatic bacteriuria (3 times),
- Intermittent preventive malaria treatment in endemic areas

We are interested in estimating the potential cost and health outcomes that can be expected from moving from the current recommendations to the new WHO antenatal recommendations.

**Section 1: Health outcomes from new WHO recommendations**

**1.1 Effect of new WHO recommendations on causes of perinatal mortality**

The current rate of perinatal mortality in Rwanda is 29 deaths per 1,000 pregnancies (year 2014/2015, source: DHS 2014/2015). For your recall, the perinatal period starts from 28 weeks of gestation, and ends seven days after birth. Therefore **perinatal mortality** is the number of stillbirths and deaths in the first week of life or early neonatal period. (4).

Below are causes of perinatal deaths in six low-income countries as reported by Ngoc et al (5). We are interested to know which of these causes you think would be affected by shifting from the current recommendations to the new WHO recommendations. Please indicate the likely percentage change (+ or -) in perinatal mortality for each cause. If you think some of the causes would not be affected, please put 0%.

|  | **Causes of perinatal deaths** | **Estimated percentage change in perinatal mortality from the following causes, if the new recommendations are implemented in Rwanda** |
| --- | --- | --- |
| 1. | Hypertensive disorders  *Troubles de l’ hypertension* |  |

| 2. | Spontaneous preterm labour  *Travail prématuré spontané* |  |
| --- | --- | --- |

| 3. | Fetal abnormalities  *Anomalies fœtales* |  |
| --- | --- | --- |
| 4. | Intrauterine fetal death  *Mort fœtale intra-utérine* |  |
| 5. | Intrapartum-related causes  *Les causes liées a l’accouchement* |  |
| 6. | Intrauterine growth restriction  *Restriction de croissance intra-utérine* |  |
| 7. | Infections  *Infections* |  |

| 8. | Maternal disease  *Maladie maternelle* |  |
| --- | --- | --- |
| 9 | Antepartum haemorrhage  *Hémorragie ante-partum* |  |

**1.2 Effect of new WHO recommendations on causes of maternal mortality**

The current rate of maternal mortality in Rwanda is 210 deaths per 100,000 live births (year 2014/2015, source: DHS 2014/2015). For your recall, according to the World Health Organization, Maternal death is the death of a woman while pregnant or within 42 days of termination of pregnancy, irrespective of the duration and site of the pregnancy, from any cause related to or aggravated by the pregnancy or its management but not from accidental or incidental causes(http://www.who.int/healthinfo/statistics/indmaternalmortality/en/)

Below are the causes of maternal deaths in Rwanda as reported by Sayinzoga et al (6). We are interested to know which of these causes you think would be affected by shifting from the current recommendations to the new WHO recommendations. Please indicate the likely percentage change (+ or -) for each cause that you think would be affected. If you think some of the causes would not be affected, please put 0%.

|  | **Cause of maternal deaths** | **Estimated percentage change in maternal mortality from the following causes, if the new recommendations are implemented in Rwanda.** |
| --- | --- | --- |
| 1. | Post-partum haemorrhage  *Hémorragie post-partum* |  |
| 2. | Obstructed labour  Travail avec obstruction / dystocie |  |
| 3. | Obstetric infection  *Infection obstétrique* |  |
| 4. | Eclampsia  *Éclampsie* |  |
| 5. | Abortion  *Avortement* |  |
| 6. | Anaesthesia complication  *Complication d'anesthésie* |  |
| 7. | Intra-partum hemorrhage  *Hémorragie intra-partum* |  |
| 8. | Amniotic embolism  *Embolie amniotique* |  |
| 9. | Abnormal pregnancy  *Grossesse anormale* |  |
| 10. | Ante-partum haemorrhage  *Hémorragie ante-partum* |  |
|  | **Other indirect causes** |  |
| 11 | Malaria  *Malaria* |  |
| 12. | AIDS  *SIDA* |  |
| 13. | Non-obstetric infection  *Infection non obstétrique* |  |
| 14. | Cardiac failure  *L'insuffisance cardiaque* |  |
| 15. | Anaemia  *Anémie* |  |
| 16. | Pulmonary embolism  *Embolie pulmonaire* |  |
| 17. | Gynaecological cancer  *Cancer gynécologique* |  |

**1.3 Overall effect of new WHO recommendations on mortality**

Please now try to estimate how much overall maternal and perinatal mortality would change as a result of shifting from the current antenatal care recommendations to the new WHO recommendations.

On a scale from 0 to 100, indicate the value corresponding to the **percentage** changes in maternal and perinatal mortality that can be expected from the change from the current antenatal care recommendations to the new WHO recommendations.

Do it by marking on the scale.

Estimate those change assuming that other factors including quality of the services, implementation arrangements, service utilization etc. do not change from the current level.

1. **Changes in perinatal mortality**

-100 -90 -80 -70 -60 -50 -40 -30 -20 -10 0 10 20 30 40 50 60 70 80 90 100

**b. Changes in maternal mortality**

-100 -90 -80 -70 -60 -50 -40 -30 -20 -10 0 10 20 30 40 50 60 70 80 90 100

**Section 2: Suggestions for improvements to current ANC recommendations**

The Government of Rwanda may adopt the new WHO ANC recommendations or may abstain. Do you think there are improvements that can be made to the current ANC model without necessarily adopting the new antenatal care model in full? Improvements can include e.g. implementation arrangements, addition of activities, or number of visits.

Please list maximum 4 suggested modifications:

1.

2.

3.

4.

What investments will be required to implement your suggested improvements? (E.g. training, purchase of equipment, recruitment of staff).

1.

2.

3.

4.

**Section 3: Potential health outcomes related to improvements in antenatal care**

Please help us to estimate potential changes in maternal and perinatal mortality that would be expected from shifting from the current antenatal care recommendations to the new improved antenatal care model (according to your suggestions).

On a scale from 0 to 100, show the values corresponding to **percentage** changes in maternal and perinatal mortality that can be expected from the change from the current antenatal care recommendations to the new model based on changes you suggested.

Do it by marking on the scale and write down the figure.

Estimate those change assuming that other factors are unchanged.

1. **Changes in perinatal mortality**

 -100 -90 -80 -70 -60 -50 -40 -30 -20 -10 0 10 20 30 40 50 60 70 80 90 100

1. **Changes in maternal mortality**

 -100 -90 -80 -70 -60 -50 -40 -30 -20 -10 0 10 20 30 40 50 60 70 80 90 100

**Thank you**

**Contact information:**

**Names:** HITIMANA Regis

**Assistant Lecturer**

**University of Rwanda, College of Medicine and Health Sciences,**

**School of Public Health**

**Email**: [regis.hitimana@gmail.com](mailto:regis.hitimana@gmail.com)

**Tel:** 0788 528 533

**REFERENCE**

1. National Institute of Statistics of Rwanda (NISR) [Rwanda], Ministry of Health (MOH) [Rwanda] and I, International. Rwanda Demographic and Health Survey. 2011;(December).

2. National Institute of Statistics of Rwanda (NISR) [Rwanda], Ministry of Health (MOH) [Rwanda] II. Rwanda Demographic and Health Survey 2014/2015. 2015.

3. Ministry of Health [Rwanda]. Success Factors for Women ’ s and Children ’ s Health: Rwanda [Internet]. 2014. Available from: http://www.who.int/entity/pmnch/knowledge/publications/nepal_country_report.pdf?ua=1

4. Allanson ER, Muller M, Pattinson RC. Causes of perinatal mortality and associated maternal complications in a South African province: challenges in predicting poor outcomes. BMC Pregnancy and Childbirth. 2012;12:24.

5. Ngoc NTN, Merialdi M, Abdel-Aleem H, Carroli G, Purwar M, Zavaleta N, et al. Causes of stillbirths and early neonatal deaths: Data from 7993 pregnancies in six developing countries. Bulletin of the World Health Organization. 2006;84(9):699–705.

6. Sayinzoga F, Bijlmakers L, van Dillen J, Mivumbi V, Ngabo F, van der Velden K. Maternal death audit in Rwanda 2009-2013: a nationwide facility-based retrospective cohort study. BMJ open. 2016;6(1):e009734.
